# From direct engagement to technical support: a programmatic evolution to improve large community health worker programs in Bihar, India

**DOI:** 10.1136/bmjgh-2020-004389

**Published:** 2021-04-14

**Authors:** Jess Wilhelm, Tanmay Mahapatra, Aritra Das, Sunil Sonthalia, Sridhar Srikantiah, Christine Galavotti, Hemant Shah, Andreea A Creanga

**Affiliations:** 1 Department of International Health, Johns Hopkins University Bloomberg School of Public Health, Baltimore, Maryland, USA; 2 CARE India Solutions for Sustainable Development, Patna, Bihar, India; 3 CARE USA, Atlanta, Georgia, USA

**Keywords:** health services research, maternal health, community-based survey, public health, epidemiology

## Abstract

**Introduction:**

In 2011, through a multipartner Integrated Family Health Initiative (IFHI), CARE started supporting maternal and neonatal health (MNH) improvement goals in 8 of 38 districts in Bihar, India. The programme included a frontline health worker (FHW) component offering health advice through household visits and benefited from CARE’s direct engagement during IFHI, which then evolved into statewide Technical Support Unit (TSU) to the Government of Bihar in 2014.

**Methods:**

Using eight rounds of state-representative household surveys with mothers of infants aged 0–2 months (N=73 093) linked with two facility assessments conducted during 2012–2017, we assessed changes in FHW visit coverage, intensity and quality between IFHI and TSU phases. Using logistic regression models, we ascertained associations between FHW outputs and three MNH core practices: ≥3 antenatal care check-ups (ANC3+), institutional delivery and early breastfeeding initiation.

**Results:**

Women’s receipt of 1+ FHW visits declined from 60.2% (IFHI phase) to 46.3% (TSU phase) in the eight IFHI districts, being below 40% statewide during the TSU phase. Despite a parallel decline in FHW visit quality measured as the number of health advice received, all three outcomes improved during the TSU versus IFHI phase in IFHI districts. We found significant positive associations between all three outcomes and receipt of 1+ FHW visits and programme phase (TSU vs IFHI) in the eight IFHI districts. During the TSU phase, receipt of 2+ FHW visits in the third trimester increased the odds of women receiving ANC3+ (adjusted OR (aOR)=1.21; 95% CI: 1.13 to 1.31), delivering in a facility (aOR=1.64; 95% CI: 1.51 to 1.77) and initiating breast feeding early (aOR=1.18; 95% CI: 1.05 to 1.18). Independent of the number and timing of FHW visits, we also found positive associations between women reporting higher than lower quality of FHW interactions and receiving outcome-specific advice and all three MNH outcomes.

**Conclusion:**

Implementation of large community-based interventions under the technical support model should be continuously and strategically evaluated and adapted.

**Key questions**

**What is already known?**

- Programmes with community-based components can accelerate improvements in maternal and neonatal health (MNH) in low-resource countries.
- In recent years, frontline health worker (FHW) programmes provided household visits and provision of health advice to pregnant and postpartum women and infants in Bihar with support from development partners, which included CARE.
- The initial, eight-district phase (Integrated Family Health Initiative, IFHI) that started in 2011 and benefited from CARE’s direct engagement evolved into a Technical Support Unit (TSU) phase in 2014.

**What are the new findings?**

- The coverage, intensity and quality of FHW services were greater during the IFHI than the TSU phase.
- During the TSU phase, receipt of 2+ FHW visits in the third trimester increased the odds of women receiving ≥3 antenatal care check-ups, delivering in a facility and initiating breast feeding early.
- Independent of the number and timing of FHW visits, we also found positive associations between women reporting higher than lower quality of FHW interactions and receiving outcome-specific advice and all three MNH outcomes.

**What do the new findings imply?**

- Given continuous improvements in MNH outcomes in Bihar, implementation of large community-based interventions under the TSU model should be continuously and strategically evaluated and adapted.

## Introduction

Community-based interventions have long been considered important components of comprehensive approaches to accelerate improvements in reproductive, maternal, newborn and child health and nutrition (RMNCHN) outcomes in low/middle-income countries (LMICs). They promote appropriate care seeking and improved home practices mainly through home-based counselling or participatory women’s groups.^1 2^ The evidence regarding the effectiveness of community-based interventions on improving such practices is strong for interventions focusing on health education, promotion of antenatal care (ANC), safe delivery with skilled birth attendants and support of exclusive breast feeding of infants.^3–5^ By and large, programmes that combine community-based and facility-based interventions are found to be more effective, suggesting a potential benefit of holistic approaches.^4 5^ The successful implementation of such programmes is expected to depend on context,^3^ thus questions remain regarding where, when and how to scale up promising interventions with a community-based component.

Walt *et al*^6^ were among the first to document common challenges with large-scale community-based programmes, including unrealistic expectations, poor initial planning, difficulties in ensuring consistency in programme quality and funding. Among commonly credited enabling factors noted by Pallas *et al*^7^ were programmes’ management structure, the inclusion of strong training and supervision components, and the integration and support from within health systems. Conversely, key barriers identified were programmes’ weak management, supervision and monitoring, coupled with inadequate incentives to retain and motivate community health workers. Smith *et al*^8^ put forth principles for successfully scaling up health interventions. Key among these were developing mechanisms to promote local ownership and building institutional capacity at all levels during implementation. In line with these principles, in recent years, we have witnessed how implementation of large health programmes placed increasing emphasis on providing technical support for programme managers, key stakeholders and governments aimed at strengthening the entire health system rather than directly engaging in specific programme activities.^9 10^ This technical support model for managers of community-based programmes is expected to channel support to community health workers despite competing programme objectives and limited resources, flexibility and capacity of non-governmental implementing partners. As noted by Chambers *et al*,^11^ over time, this model may lead either to a ‘voltage drop’ or to sustainable scale-up of pilot programmes if adequately adapted to the local context.

With a population of over 1.3 billion people, India accounts for 12% of maternal deaths^12^ and 35% of neonatal deaths worldwide.^13^ In 2005, the Government of India launched the National Rural Health Mission (NRHM) to strengthen provision of community health services and increase equity and affordability of care in rural areas.^14^ Interventions implemented under the NRHM included staffing, training, and equipping referral facilities and deploying community-based cadres, referred to as frontline health workers (FHWs), to promote institutional deliveries, particularly in low-performing Indian states.^15–18^ FHW cadres include accredited social health activists (ASHAs) and Anganwadi workers (AWWs) who have a minimum of 8 and 10 years of education, respectively, as well as auxiliary nurse midwives (ANMs) who are trained nurses. Under the NRHM, the Janani Suraksha Yojana (JSY) initiative provides 1400 Indian rupees to eligible women who deliver in health facilities as well as incentives for the ASHAs in the communities where these women reside for accompanying them to the facility or for facilitating institutional birth.^19^ With NRHM funding, large numbers of health workers, including doctors, staff nurses, ANMs and ASHAs were contracted during 2005–2014 in low-performing Indian states. Bihar, the third most populous state where >34% of the population lived below the poverty line,^20^ was one of them. The state has seen significant improvements in health indicators in recent years, such as the rise in institutional deliveries from 19.9% in 2005–2006 to 63.8% in 2015–2016,^21^ but FHW mobilisation to promote health services at the community level was particularly poor in Bihar.^22^

CARE India is a non-profit organisation and a lead partner in a multiorganisational platform aiming to improve RMNCHN in Bihar.^23^ In 2011, CARE launched the Integrated Family Health Initiative (IFHI) in eight programmatically prioritised districts of Bihar covering 28 million people to support the Government of Bihar (GoB) in increasing the coverage and quality of life-saving interventions for women, newborns and children.^23^ Programme support for IFHI involved direct engagement with programme activities by CARE (table 1). In close collaboration with the GoB, CARE and other partners participated in planning, coordination and logistical support; capacity building; behaviour change communications; work with women’s self-help groups (SHGs) and health system strengthening. For community-level interventions, CARE employed an incremental learning approach, involving FHWs through the existing 2550 health subcentre platforms. The main IFHI hypothesis was that an increase in the level and quality of efforts on the part of FHWs would result in improved service coverage in facilities as well as improvements in healthcare practices and behaviours at the family level. A close connect to the family in the last trimester of pregnancy was strongly emphasised as critical to a range of outcomes for the mother and baby. The IFHI phase generated convincing evidence to take interventions to scale.^23^

**Table 1:** Intervention phases in Bihar, India

Integrated family health initiative (2011–2013)	Technical support unit (2014–2017)
*Direct engagement in programme activities*	*Technical support*
Participate in planning and coordination of programme activities in eight initial districts Direct provision of logistical support Direct involvement with capacity building in health facilities and for government cadres Direct supervision of programme staff Health system strengthening (eg, facility infrastructure, supply chains and information systems improvements) Engagement with FHWs employing an incremental learning approach and using the health subcentre platform offering: didactic sessions gradually introducing new topics Mobile Kunji as job aids for messaging data to demonstrate programme gaps home visit planners to ensure timely contacts and relevant messaging by women’s pregnancy trimester Behavioural change communications Work with women’s self-help groups	Participate in planning and coordination of programme activities at scale in all 38 districts Capacity building for block-level leadership and government cadres Strengthening of overall supervisory system for the programme Focused efforts on select interventions (eg, nurse mentoring and training) Engagement with FHW supervisors rather than FHWs Technical assistance for behavioural change communications Support of women’s self-help groups during state planning

FHWs, frontline health workers.

During 2014, in close coordination with GoB, the programme evolved into a Technical Support Unit (TSU) and was rolled out in all 38 districts of Bihar covering over 104 million people. This phase was characterised by a considerable reduction in direct engagement in programme activities by CARE, yet deeper focus on capacity building, strengthening the supervisory system, synergising and focusing efforts on select interventions (eg, nurse mentoring and training),^24^ and using monitoring data. As a result, CARE acted as a catalyst to facilitate provision of necessary technical, managerial and resource optimisation support at critical levels of programme implementation, while building community and government ownership of the programme. With regard to the FHWs, the shift from IFHI to TSU can be characterised as a shift from relatively greater direct engagement to relatively greater technical support.

This analysis focuses on the FHW component of the programme in Bihar during 2011–2017. We aimed to: (1) assess and compare the coverage, intensity, and quality of FHWs’ interactions with pregnant women during household visits during IFHI (eight districts) and TSU (statewide) phases, and (2) ascertain and compare associations between FHW programme outputs and the uptake of three core maternal and neonatal health (MNH) programme practices: ≥3 ANC check-ups (ANC3+), institutional delivery and early (<1 hour of birth) breastfeeding initiation. Of note, early initiation of breast feeding was a key programme target in Bihar during both IFHI and TSU phases; ANC and institutional delivery became specific programme targets only in 2015 and 2016, respectively, after the programme started to benefit from a multidimensional quality improvement initiative implemented in all public health facilities in the state between 2014 and 2017.^24^

## Methods

### Patient and public involvement

Women and their families were not directly involved in setting the research questions or the outcome measures for the programme or this specific analysis of programme data. However, these were developed in consultation with and guidance from community leaders, and women and their families were intimately involved in the design and implementation of the programme in Bihar. Through community sensitisation meetings, they received information about the programme and its goals, which in turn helped motivate their participation in household surveys. We intend to disseminate the main results of this analysis to the community in Bihar through CARE India colleagues who continue to be engaged in the programme to serve the community.

### Data

We used eight rounds of household surveys conducted by CARE with mothers of infants 0–2 months old in Bihar. Four survey rounds (P1–P4) were conducted about 6 months apart during the IFHI phase (2012–2013) in all 137 blocks in eight districts using lot quality assurance sampling methodology; the total P1–P4 sample was 10 408 mothers (online supplemental table 1). Systematic random samples of mothers, 1 from each of 19 randomly selected Anganwadi Center (AWC) areas in each block, were recruited for interviews; weights were derived using the number of births in each block to generate rural population-based estimates of intervention coverage and outcomes of interest. Four household survey rounds (S1–S4) were conducted statewide annually during the TSU phase (2014–2017) using a two-stage stratified sampling methodology covering all 534 blocks in all 38 districts; the total S1–S4 sample was 62 685 mothers. Within each block, the AWC list from GoB’s Integrated Child Development Scheme was used as sampling frame, being considered the most comprehensive and up-to-date list.^25^ Samples were distributed across different blocks to obtain a minimum sample size of 388 mothers per district and 19 mothers per block. The number of AWCs selected in each block was determined based on the proportional contribution of each block’s population to the respective district population, aiming to sample one household with infants 0–2 months old in each selected AWC. Within each AWC, the household list compiled by the local AWW was used to randomly select an index household in the community. The index household was not sampled, and enumerators proceeded to the fifth household from the index in a clockwise fashion to identify resident mothers of infants 0–2 months old who had delivered more than 7 days before. If the first fifth household did not have an eligible mother present, the enumerator continued to the following fifth household until an eligible mother was identified for interview. Block-level survey weights were generated to account for the complex survey design and were used in our analyses. Verbal informed consent was obtained from eligible women who agreed to complete the interviews. District-level response rates varied between 96% and 98% across survey rounds.

10.1136/bmjgh-2020-004389.supp1 Supplementary data

Survey questionnaires collected information on women’s sociodemographic characteristics, obstetric history, JSY programme participation, interactions with FHWs and the types of health advice received from them during and after pregnancy, receipt of ANC and delivery care as well as neonatal care practices. While some variation between survey questionnaires existed for P1–P4 vs S1–S4 surveys, for this analysis, we selected questions that were consistently asked across survey rounds to derive FHW and outcome indicators of interest.

### Analyses

We generated FHW programme output variables: a binary (yes/no) indicator to identify women who received 1+ FHW visits during pregnancy; a four-category variable derived from three survey questions (‘At any time during the pregnancy, did (ASHA/AWW/ANM) ever come to your home to talk to you about your or your expected baby’s health?’, ‘Was it a health-related visit?’, and ‘How many visits did you receive from FHWs during the third trimester of pregnancy?’) and categorised as: received no FHW visits during pregnancy, received 1+ FHW visit(s) but not in the last trimester of pregnancy, received only 1 FHW visit during the last trimester of pregnancy, and received 2+ FHW visits during the last trimester of pregnancy; indicators capturing specific types of FHW advice (ie, danger signs, institutional delivery, delivery with a skilled birth attendant, safe delivery practices, early breast feeding, skin-to-skin care, family planning); and an index of the quality of interactions with FHWs derived from questions regarding advice received across five domains: birth preparedness for institutional delivery, birth preparedness for home delivery, recognition and care seeking for maternal complications, essential newborn care practices and postpartum family planning. This quality index accounts for the number of domains of advice received (0–5 range). For ease of interpretation, in the analysis, we used a binary FHW interaction quality variable categorised as ‘high quality’ if the index is ≥4 (90th percentile of index score) and ‘average or lower quality’ if the index is <4.

We explored trends in several outcomes: ANC (any, 3+, 4+ check-ups); institutional delivery; presence of skilled birth attendant for home deliveries; skin-to-skin practice right after birth; early initiation of breast feeding, defined as breast feeding initiated <1 hour after birth and estimated only among women with a vaginal delivery; and newborn weighing. For the analysis of FHW visits’ effects, we selected three outcomes based on their relevance in the MNH field and the availability of strong evidence around community-based interventions’ success on improving these outcomes^4 5^: ANC3+, institutional delivery and early initiation of breast feeding. A fourth outcome, television ownership, was chosen as ‘falsification test’^26^ to assess potential confounding in the associations of interest between FHW outputs and outcomes. We suspected that latent factors, such as women’s willingness to adopt new behaviours, may result in both more efficient interactions with FHWs and improved outcomes. But, it is unlikely that household visits and provision of health advice by FHWs would lead to a television purchase, selected over other types of purchases because of its lower prevalence than other household items (eg, radio) in Bihar and because it is purely a consumption good, unlike vehicles or gas stoves.

We identified potential confounders of the associations of interest. Sociodemographic factors included maternal age, parity, religion, caste, mother’s and husband’s education and literacy, tertiles of a household wealth index generated as time-varying price-weighted sum of 25 household items (P1–P4) and 27 items (S1–S4) assessed in each survey round. Contextual factors included: JSY programme participation (yes/no) available in all survey rounds, and SHG membership (yes/no) only available in S1–S4. Facility-level factors in block-level facilities where the woman resided were obtained from two rounds of comprehensive facility assessments conducted in 2015 (linked with P1–P4 and S1 data) and 2016 (linked with S2–S4 data). They included the level of block-level facility (lowest level considered if more than one facility per block as most likely first source of care), and the ratio of clinical staff (doctors, nurses, nurse-midwives) filled to that approved in the block-level facility.

For our first study objective, we first examined trends in FHW programme outputs by survey round and inclusion in the IFHI phase, and trends in outcomes by survey round and place of delivery (ie, home, public or private facility) when applicable. Multivariable logistic regression models were fitted for each of the outcomes using only the data from the eight IFHI districts comparing the TSU with the IFHI phase. Models were adjusted for women’s receipt of 1+ FHW visits, an interaction term between programme phase and receipt of 1+ FHW visits, and the potential confounders listed above; the model fitted for early breastfeeding initiation was also adjusted for whether the woman delivered in a facility or not. Models were estimated using generalised estimating equations to account for clustering of facility-level covariate data. In regression analyses, we controlled for all potential confounders noted above.

For the second study objective, we fitted a second set of four multivariable logistic regression models using only the TSU phase (S1–S4) data. Models were adjusted for the FHW household visit frequency, the quality of interactions with FHWs, the provision of outcome-specific advice as well as the potential confounders listed above. Compared with the first set of regression models, these models were adjusted for SHG membership and IFHI district status. Models were estimated using generalised estimating equations to account for clustering of facility-level covariate data. Using this second set of regression models, we estimated the predicted marginal probabilities of the outcomes under a variety of hypothetical scenarios to illustrate the relative impact of FHW visit frequency, quality of interactions and provision of outcome-specific advice compared with the impact of changes in other factors known to influence MNH outcomes: female literacy, SHG membership and delivery in a health facility.

All analyses were conducted in Stata V.15 and accounted for the complex survey design using Taylor’s linearisation method. Data can be made available upon request and under a data use agreement with CARE, India.

## Results

About three in four women in our samples were 20–29 years old and had two or three children (table 2). The majority of women were Hindu and belonged to the non-marginalised caste. Among women interviewed during the IFHI phase, 36.4% were literate; this percentage was higher (44.6%) for women interviewed during the TSU phase.

**Table 2:** Population characteristics by programme phase: Bihar, 2011–2017

Characteristics	Initial eight programme districts		Additional 30 districts	All 38 districts
	*IFHI*	*TSU*	*TSU*	*TSU*
N	10 408	13 767	48 918	62 685
Age (years; %)				
<20	6.0	11.3	9.7	10.1
20–24	42.9	45.5	46.3	46.1
25–29	33.4	29.8	30.4	30.2
30–34	12.8	9.8	9.8	9.9
35+	5.0	3.7	3.9	3.9
Mean (SE; years)	24.9 (0.5)	24.1 (0.4)	24.3 (0.2)	24.2 (0.2)
Religion (%)				
Hindu	86.5	86.0	83.2	84.1
Other*	13.5	14.0	16.8	15.9
Caste (%)				
Marginalised (Scheduled Caste/Tribe)	26.8	24.7	27.7	27.0
Non-marginalised	73.2	75.3	72.3	73.0
Household wealth index†				
Lowest tertile	34.0	31.0	34.3	33.4
Middle tertile	33.2	36.1	33.4	34.1
Highest tertile	32.8	32.8	32.3	32.5
Knows to read and write (%)	36.4	43.6	45.0	44.6
Education (completed years; %)				
No formal education	63.6	56.7	55.8	56.1
1–8	21.3	21.3	19.5	20.0
>8	15.1	21.9	24.7	24.0
Husband knows to read and write (%)	58.5	60.5	60.2	60.2
Husband’s education (completed years; %)				
No formal education	42.9	44.2	43.8	43.9
1–8	31.0	27.2	23.4	24.
>8	26.0	28.6	32.8	31.7
Mean (SE) number of living children	2.8 (0.2)	2.8 (0.2)	2.7 (0.1)	2.7 (0.1)
Mean (SE) age of last born at the time of interview (days)	42 (0.3)	47 (0.3)	47 (0.1)	47 (0.1)

All data are weighted.

*Includes Muslim, Christian, other religion.

†Household wealth index generated as time-varying price-weighted sum of 25 (IFHI phase) or 27 (scale-up phase) household items assessed.

IFHI, Integrated Family Health Initiative; TSU, Technical Support Unit.

### Coverage of FHW interactions with pregnant women

In the eight IFHI districts, the coverage of pregnant women with 1+ FHW visits during pregnancy peaked at 65.0% in round P4 at the end of the IFHI phase but declined during the TSU phase to about 40% (p<0.001); this level of coverage was above that in the 30 non-IFHI districts, which was stagnant at <40% until the end of 2017 (table 3, online supplemental table 2). Coverage of 2+ visits during third trimester of pregnancy declined even more dramatically from 60.2% in round P4 to 24.1% in round S4 (p<0.001) in the eight IFHI districts, being at <30% in the 30 non-IFHI districts in rounds S2–S4. Trends in FHWs offering specific types of advice, including advice regarding institutional delivery and early breastfeeding initiation, followed similar patterns. The quality of interactions with FHWs during household visits also declined significantly (p<0.001) from an average index score of 3.3 domains of advice covered by FHWs at the end of the IFHI phase (P4) to around 2 domains of advice covered in the eight IFHI districts during the TSU phase (online supplemental figure 1). Declines in advice related to home birth preparedness, maternal complications and related care seeking, and postpartum family planning appear to be driving this trend.

**Table 3:** Summary of contextual, output and outcome measures by programme phase: Bihar, 2011–2017

Measures	Initial eight programme districts		Additional 30 districts	All 38 districts
	*IFHI*	*TSU*	*TSU*	*TSU*
N	10 408	13 767	48 918	62 685
Contextual				
JSY programme participation (%)	38.7	12.4	14.6	14.0
Self-help group membership (%)	n/a	15.6	15.4	15.5
FHW programme outputs				
FHW household visit frequency (%)				
No visit during pregnancy	39.8	53.7	61.5	59.3
Only visits in 1st or 2nd pregnancy trimester	1.3	6.8	4.9	5.4
Only one visit during 3rd pregnancy trimester	5.5	8.4	7.0	7.4
2+ visits during 3rd pregnancy trimester	53.4	31.2	26.6	27.8
Specific advice (%)				
Advice for institutional delivery	46.9	36.9	31.4	32.9
Advice for early initiation of breast feeding*	41.2	22.9	16.1	18.0
Quality of FHW interactions during household visits† (%)				
Received advice on 4+ domains	43.4	21.9	18.2	19.4
Outcomes for this analysis				
Attended 3+ ANC visits (%)	22.3	40.9	43.2	42.6
Institutional delivery (%)	72.6	75.6	70.9	72.2
Early initiation of breast feeding* (%)	57.1	70.1	66.2	67.2
TV ownership (%)	12.4	15.6	17.5	17.0

All data are weighted.

The index accounts for the number of domains of advice received across five domains: birth preparedness for institutional delivery, birth preparedness for home delivery (whether planned or unplanned), recognition and care seeking for maternal complications, essential newborn care practices and postpartum family planning. The index ranges between 0 and 5.

*Early initiation of breast feeding refers to initiation within the first hour after birth and is only measured among women who had a vaginal delivery.

†The quality index was estimated among mothers of infants aged 0–2 months old who received one or more FHW visits during pregnancy.

ANC, antenatal care; FHW, frontline health worker; IFHI, Integrated Family Health Initiative; JSY, Janani Suraksha Yojana; n/a, not available; TSU, Technical Support Unit.

### Changes in MNH outcomes during the programme

Despite declines in FHW outputs, we found a significant increase in ANC3+ between the time of the S1 survey and the period captured by S2–S4 surveys, more so among the non-IFHI than IFHI districts (p<0.001; online supplemental table 3). Institutional deliveries remained about constant over time, with a slight increase noted at the time of the S4 survey and levels being overall higher among IFHI than non-IFHI districts during the TSU phase. Early initiation of breast feeding increased in the IFHI districts throughout the initial phase, reached a peak at the time of the S2 survey (p<0.001), to decline afterwards as captured in S3 and S4 surveys; levels for this outcome indicator were slightly lower in non-IFHI than IFHI districts throughout the TSU phase.

### Changes in FHW programme outputs and MNH outcomes between programme phases

We compared FHW programme outputs, MNH outcomes, and contextual measures between the IFHI and the TSU phase in the eight IFHI districts, and between the 8 IFHI vs 30 non-IFHI districts during the TSU phase. While, on average, 53.4% of pregnant women in the eight IFHI districts received 2+ FHW visits during the third trimester of pregnancy during the IFHI phase, the proportion declined to 31.2% during the TSU phase (p<0.001); only 26.6% of pregnant women received such visits in the 30 non-IFHI districts during the TSU phase (table 3). Of women who received a FHW visit, the proportion with high-quality FHW interactions (ie, quality index ≥4 domains of advice) declined from 43.4% (IFHI phase average) to 21.9% (TSU phase average) in the eight IFHI districts (p<0.001), and was 18.2% (TSU phase average) in the 30 non-IFHI districts. Coverage of specific advice for institutional delivery declined from 46.9% to 36.9% (p<0.001) in the eight IFHI districts between the IFHI and TSU phases, being 31.4% for the 30 non-IFHI districts during the TSU phase. The decline in coverage of FHW visits with advice for early breastfeeding initiation was even more pronounced dropping from an average of 41.2%–22.9% in the eight IFHI districts between the IFHI and TSU phases, being 16.1%, on average, in the 30 non-IFHI districts during the TSU phase. Despite declining coverage and quality of FHW interactions, outcomes improved during the TSU phase compared with the IFHI phase (22.3%–40.9% for ANC3+, 72.6%–75.6% for institutional delivery, and 57.1%–70.1% for early breastfeeding initiation) in the eight IFHI districts. Also, participation in the JSY programme declined considerably between the initial (38.7%) and the TSU (12.4%) phase in the eight IFHI districts, being 14% statewide during the TSU phase (p<0.001). Membership in SHG was 15.5% statewide during the TSU phase.

### Associations between FHW visits and MNH outcomes in the initial eight districts by programme phase

After adjustment for potential confounders, we found significant positive associations between the three MNH outcomes (ANC3+, institutional delivery and early initiation of breast feeding) and receipt of 1+ FHW visits and programme phase (TSU vs IFHI) in IFHI districts (table 4). Delivery in a facility increased the odds of initiating breast feeding early (adjusted OR (aOR)=2.24, 95% CI: 2.10 to 2.40). Women’s and their husbands’ literacy were positively associated with attendance of ANC3+ check-ups and institutional delivery, but not with early initiation of breast feeding. Women of higher parity and those belonging to marginalised than non-marginalised caste had lower odds of attending ANC3+ check-ups and delivering in a facility, but higher odds of initiating breast feeding early. Having a higher level facility than only a primary health centre in the block was associated with higher odds of having ANC3+ check-ups, while higher filled-to-approved clinical staffing ratios were associated with higher odds of ANC3+ check-ups and institutional delivery.

**Table 4:** Results from multivariable logistic regression models fitted for selected programme outcomes and a control outcome: Bihar, 2011–2017

Covariates	IFHI and TSU phase data in the initial eight programme districts*				TSU phase data in all 38 districts†			
	Institutional delivery	ANC3+	EIBF	TV ownership	Institutional delivery	ANC3+	EIBF	TV ownership
	Adjusted OR (95% CI)							
**Sociodemographic characteristics**								
Age	1.006 (0.997 to 1.016)	1.018*** (1.008 to 1.028)	0.991 (0.981 to 1.001)	1.042*** (1.027 to 1.058)	1.011*** (1.005 to 1.017)	1.033*** (1.027 to 1.039)	0.992* (0.986 to 0.998)	1.044*** (1.035 to 1.052)
Parity	0.854*** (0.832 to 0.876)	0.826*** (0.803 to 0.850)	1.050*** (1.023 to 1.077)	0.880*** (0.844 to 0.919)	0.842*** (0.829 to 0.855)	0.775*** (0.762 to 0.789)	1.047*** (1.030 to 1.065)	0.871*** (0.850 to 0.892)
Non-Hindu religion (Hindu=ref)	0.866** (0.788 to 0.952)	1.162** (1.061 to 1.273)	0.648*** (0.594 to 0.709)	0.718*** (0.621 to 0.830)	0.743*** (0.702 to 0.786)	1.141*** (1.079 to 1.205)	0.685*** (0.648 to 0.724)	0.736*** (0.678 to 0.800)
Marginalised caste (other=ref)	0.703*** (0.655 to 0.754)	0.737*** (0.685 to 0.794)	1.128** (1.052 to 1.210)	0.610*** (0.539 to 0.689)	0.602*** (0.577 to 0.629)	0.722*** (0.692 to 0.753)	1.104*** (1.056 to 1.154)	0.641*** (0.601 to 0.683)
Middle wealth index tertile (high index tertile=ref) Low index tertile	1.061 (0.985 to 1.142) 1.092* (1.011 to 1.179)	0.874*** (0.812 to 0.940) 0.927* (0.862 to 0.997)	1.011 (0.942 to 1.086) 0.970 (0.902 to 1.044)	0.101*** (0.078 to 131) 1.778*** (1.616 to 1.957)	1.045 (0.999 to 1.094) 1.031 (0.985 to 1.078)	0.934** (0.895 to 0.975) 0.960 (0.920 to 1.002)	1.014 (0.969 to 1.061) 0.951* (0.909 to 0.995)	0.092*** (0.083 to 0.103) 0.792*** (0.752 to 0.835)
Woman’s literacy (no=ref)	1.624*** (1.507 to 1.751)	1.704*** (1.595 to 1.821)	1.000 (0.988 to 1.070)	2.75*** (2.473 to 3.077)	1.587*** (1.518 to 1.659)	1.650*** (1.586 to 1.717)	0.983 (0.941 to 1.026)	2.664*** (2.508 to 2.830)
Husband’s literacy (no=ref)	1.224*** (1.145 to 1.309)	1.384*** (1.290 to 1.477)	0.968 (0.907 to 1.032)	2.337*** (2.063 to 2.648)	1.296*** (1.244 to 1.351)	1.437*** (1.381 to 1.496)	1.011 (0.970 to 1.055)	2.521*** (2.353 to 2.702)
**Context variables**								
Delivered in facility (no=ref)			2.244*** (2.097 to 2.401)				2.601*** (2.494 to 2.713)	
TSU phase (IFHI=ref)	1.460*** (1.143 to 1.366)	2.384*** (2.159 to 2.632)	1.514*** (1.388 to 1.651)	1.504*** (1.310 to 1.727)				
Data round S1 (S4=ref) S2 S3					0.82*** (0.775 to 0.868) 0.713*** (0.676 to 0.752) 0.702*** (0.666 to 0.740)	0.192*** (0.181 to 0.203) 0.687*** (0.655 to 0.720) 0.792*** (0.757 to 0.830)	0.595*** (0.564 to 0.628) 1.375*** (1.302 to 1.452) 0.791*** (0.752 to 0.833)	0.573*** (0.532 to 0.618) 0.759*** (0.710 to 0.812) 0.781*** (0.732 to 0.834)
IFHI district (no=ref)					1.230*** (1.095 to 1.380)	0.838*** (0.757 to 0.926)	1.089 (0.999 to 1.188)	0.864** (0.778 to 0.960)
Self-help group membership (no=ref)					1.111*** (1.054 to 1.170)	1.035 (0.986 to 1.087)	1.129*** (1.070 to 1.192)	0.988 (0.919 to 1.063)
**Frontline health worker (FHW) programme outputs**								
1+ household visits (no visit=ref)	1.743*** (1.586 to 1.916)	1.197*** (1.077 to 1.330)	1.221*** (1.118 to 1.333)	0.975 (0.841 to 1.130)				
Interaction term for 1+ FHW visits and programme phase	0.978 (0.864 to 1.107)	0.968 (0.853 to 1.099)	1.352*** (1.201 to 1.522)	0.961 (0.800 to 1.153)				
Household visit frequency (no visit=ref) 1st/2nd trimester visits only OR 1 visit in 3rd trimester 2+ visits in 3rd trimester					1.191*** (1.099 to 1.291) 1.637*** (1.510 to 1.773)	0.968 (0.898 to 1.043) 1.212*** (1.126 to 1.305)	1.113*** (1.048 to 1.182) 1.183*** (1.121 to 1.248)	1.020 (0.919 to 1.131) 0.983 (0.886 to 1.092)
High quality of interactions with FHWs (low/average quality=ref)					1.103* (1.012 to 1.202)	1.226*** (1.142 to 1.318)	1.244*** (1.133 to 1.365)	1.127* (1.012 to 1.254)
Outcome-specific advice (not received=ref) Institutional delivery Early initiation of breast feeding					1.181*** (1.091 to 1.279)	1.183*** (1.100 to 1.272)	1.678*** (1.562 to 1.802)	0.909 (0.821 to 1.006) 1.093 (1.000 to 1.195)
**Facility factors**								
First referral unit facility (primary health centre=ref) District hospital	1.015 (0.733 to 1.407) 0.997 (0.766 to 1.297)	1.907*** (1.365 to 2.664) 1.158 (0.888 to 1.570)	1.018 (0.850 to 1.219) 0.910 (0.776 to 1.069)	1.744*** (1.349 to 2.254) 1.941*** (1.543 to 2.442)	1.061 (0.906 to 1.243) 1.202* (1.026 to 1.410)	1.207** (1.047 to 1.391) 1.198* (1.042 to 1.378)	0.961 (0.852 to 1.084) 0.854** (0.758 to 0.962)	1.067 (0.928 to 1.228) 1.278*** (1.114 to 1.465)
Clinical staff filled: approved ratio (fully staffed=ref)	1.020 (0.959 to 1.085)	1.267*** (1.198 to 1.340)	1.057 (0.999 to 1.117)	1.098* (1.019 to 1.183)	1.060** (1.018 to 1.105)	1.061*** (1.024 to 1.100)	0.950** (0.919 to 0.983)	1.077*** (1.035 to 1.121)
*Constant*	2.205*** (1.705 to 2.851)	0.130*** (0.099 to 0.172)	0.760* (0.599 to 0.965)	0.0164*** (0.011 to 0.025)	2.578*** (2.200 to 3.020)	0.609*** (0.524 to 0.707)	1.201* (1.030 to 1.400)	0.060*** (0.049 to 0.074)

Adjusted ORs are statistically significant at *p<0.05; **p<0.01 or ***p<0.001.

*Models fitted for the four outcomes listed adjusting for programme phase (TSU vs IFHI used as reference), for all the other factors shown, and for clustering at the block level using only data from the initial eight programme districts.

†Models fitted for the four outcomes listed adjusting for TSU programme phase (S1–S3 vs S4 used as reference), for all the other factors shown, and for clustering at the block level using only TSU phase data for all 38 districts.

ANC, antenatal care; EIBF, early initiation of breast feeding; IFHI, Integrated Family Health Initiative; S1–S4, data round during TSU phase; TSU, Technical Support Unit.

### Associations between FHW visits and MNH outcomes in all 38 districts during the TSU phase

During the TSU phase, compared with women in the 30 non-IFHI districts, those in the eight IFHI districts had higher odds of delivering in a health facility (aOR=1.23; 95% CI: 1.09 to 1.38), yet lower odds of having ANC3+ check-ups (aOR=0.84; 95% CI: 0.76 to 0.93; table 4). Also during the TSU phase, receipt of 2+ FHW visits in the third trimester increased the odds of women receiving ANC3+ (aOR=1.21; 95% CI: 1.13 to 1.31), delivering in a facility (aOR=1.64; 95% CI: 1.51 to 1.77) and initiating breast feeding early (aOR=1.18; 95% CI: 1.05 to 1.18). Importantly, independent of the number and timing of FHW visits received, we found positive associations between women reporting a higher quality of interactions with FHWs and all ANC3+, institutional delivery and early initiation of breast feeding; in addition, receipt of outcome-specific advice (eg, to deliver in a facility) increased the odds of all three MNH outcomes, especially early breastfeeding initiation. Interestingly, SHG membership was positively associated with institutional delivery and early initiation of breast feeding, but not ANC3+. Associations between sociodemographic characteristics and all ANC3+, institutional delivery and early initiation of breast feeding were of similar magnitude and significance in models fitted for all 38 districts during the scale-up period as for the pooled initial and scale-up data from the eight IFHI districts. During the TSU phase, having a higher level than only a primary health centre in the block was associated with higher odds of women obtaining ANC3+ check-ups and delivering in a facility, but lower odds of early initiation of breast feeding.

### Changes in MNH outcomes under hypothetical FHW or other programme scenarios

Based on observed variable associations at the state level during the TSU phase, if all women would receive 2+ FHW visits in the third trimester of pregnancy with high-quality interactions and outcome-specific advice, the percentage of women with ANC3+ check-ups would increase from 41.7% to 48.5%, that of women delivering in health facilities from 73.5% to 82.2%, and that of women initiating breast feeding early from 67.5% to 81.9% (table 5). This scenario would lead to higher percentage point increases in coverage of all ANC3+, institutional delivery and early initiation of breast feeding than the estimated effects of achieving 100% maternal literacy rate or having all mothers in Bihar become members of SHGs.

**Table 5:** Intervention scenarios and corresponding predicted marginal probabilities of selected outcomes during the TSU phase: Bihar, 2014–2017

Intervention scenarios	Institutional delivery	ANC3+	EIBF
Predicted marginal probabilities at mean values	0.735 (0.726 to 0.744)	0.417 (0.408 to 0.426)	0.675 (0.667 to 0.682)
No woman gets FHW visits during pregnancy	0.696 (0.685 to 0.706)	0.396 (0.386 to 0.405)	0.630 (0.621 to 0.638)
All women get FHW visits during pregnancy	0.772 (0.761 to 0.784)	0.425 (0.413 to 0.438)	0.693 (0.683 to 0.702)
All women get 2+ FHW visits during third pregnancy trimester with low-to-moderate quality of interactions with FHWs, but no outcome-specific advice	0.783 (0.768 to 0.797)	0.408 (0.392 to 0.424)	0.693 (0.682 to 0.705)
All women get 2+ FHW visits during last trimester with high quality of interactions with FHWs, but no outcome-specific advice	0.798 (0.780 to 0.817)	0.450 (0.429 to 0.472)	0.735 (0.716 to 0.755)
All women get 2+ FHW visits during last trimester with high quality of interactions with FHWs and outcome-specific advice	0.822 (0.809 to 0.835)	0.485 (0.469 to 0.501)	0.819 (0.807 to 0.832)
All women get 2+ FHW visits during last trimester with high quality of interactions with FHWs and outcome-specific advice and all deliveries occur in health facility	n/a	n/a	0.849 (0.838 to 0.860)
100% deliveries in a health facility	n/a	n/a	0.733 (0.726 to 0.741)
100% mothers are members of self-help groups	0.751 (0.739 to 0.762)	0.423 (0.410 to 0.435)	0.695 (0.684 to 0.707)
100% mothers are literate	0.784 (0.774 to 0.794)	0.474 (0.463 to 0.484)	0.673 (0.664 to 0.682)

Predicted marginal probabilities are from multivariable regression models fitted using the scale-up phase data from all 38 districts and shown in table 4.

ANC, antenatal care; EIBF, early initiation of breast feeding; FHW, frontline health worker; n/a, not available; TSU, Technical Support Unit.

## Discussion

Our findings indicate that the IFHI model of intense programming and direct engagement in programme activities by CARE did not sustain its impact on FHW service coverage and interaction quality during the subsequent, less intensive, but scaled-up model of technical support in Bihar. This erosion of impact is neither surprising nor overwhelming as positive changes were indeed observed in MNH outcomes during the TSU phase above and beyond secular trends. Importantly, these outcomes were positively associated with higher quality and outcome-targeted interactions between FHWs and pregnant women in the community. Taken together, our findings in Bihar mirror the experience with other large-scale programmes in LMICs where scaling up using a ‘technical support’ model also faced challenges, despite overall positive changes in programme outcomes.^27–30^ To place our findings in the broader Indian context, a systematic review of research published between 2005 and 2016 examining India’s FHW programmes found that most articles had mixed results and only a few indicated overall positive findings.^31^

There are known reasons for which the technical support model faced challenges in achieving sustainable scale-up of the FHW programme component in Bihar. Well recognised among these, the GoB encountered difficulties in supporting AWWs during 2014–2016,^32^ but was then able to even increase their payment in 2017.^33^ However, a recent study conducted in four Indian states showed that ASHAs’ visits to beneficiaries were not more strongly associated with outcomes for which they were paid than outcomes for which they were unpaid.^34^ The Home Based Newborn Care initiative, rolled out in Bihar after 2014, provided incentives to ASHAs for postnatal follow-up visits and tracking of low birthweight newborns.^35^ The increased emphasis on postnatal care, while important, might have also affected the time ASHAs had for household visits to pregnant women. FHWs’ performance was found to reflect the quality of their training, the time lapse since their last training, the intensity of mentoring and supervision they receive, and their work motivation.^36 37^ Supervision appears to influence FHWs’ motivation in India, with government than non-governmental organisation supervisors being less effective in motivating FHWs.^36^

ANC became a programme target in Bihar only in 2015. Nonetheless, we found positive, although modest associations between all 2+ FHW visits, the quality of FHW interactions and receipt of ANC-specific advice from FHWs on women having ANC3+ check-ups. Analyses of Indian Human Development Survey data showed that exposure to ASHA services was associated with a 17% and 5% increase in women attending 1 and 4+ ANC visits, respectively.^38^ However, research in several Indian states found ANC services provided by ASHA workers were not adequate.^39^ In our study, women’s literacy and affiliation with SHGs was found to be positively associated with ANC attendance, likely through increased awareness, self-efficacy or perception of ANC as social norm. These findings have likely been influenced by the improvements in public facilities through the facility-based component of the programme over time. Another notable factor behind the rising ANC3+ trend might be the village health, sanitation and nutrition days (VHSND) platform, which made ANC interventions available to large numbers of pregnant women within the community.

Institutional deliveries increased considerably in Bihar as the JSY programme expanded during 2011–2012. With delayed payments and changes from distributing cash to making bank deposits, the proportion of mothers reporting receiving JSY programme incentives declined from 46% (P4 data) to 6% (S3 data)—the trend in institutional deliveries remained flat during this time as the programme in Bihar also prioritised improving quality of care in public facilities rather than reaching a higher institutional delivery target (online supplemental figure 2). Following an increase in JSY participation and the programme focusing on such in 2017 (S4 data), the proportion of institutional deliveries increased again. These data suggest that delivery in a health facility is closer to becoming the norm in Bihar despite changes with the JSY programme, and the FHWs appear to have played an important role in this positive trend. Women’s literacy also appears to be an important driver for institutional delivery, a result of the focus placed on girls’ education in the early 2000s.^40^ Others found similar associations between exposure to FHWs and institutional deliveries in India.^34 38 41 42^

Visits by FHWs have plausibly driven the increasing proportion of women initiating breast feeding early—they appear to, both directly and indirectly through institutional delivery, influence early breastfeeding practices. Only 18.0% (statewide TSU phase average) of pregnant women received advice from FHWs to start breast feeding early, a level that is comparable with that reported in the evaluation of a similar programme in Uttar Pradesh.^43^ We found that if all women received 2+ FHW visits in the third trimester that are of high quality and include advice for early breast feeding, the predicted coverage of early breast feeding would increase from 67.5% to 81.9%, or even to 84.9% if all deliveries would also occur in health facilities.

This study has several limitations. We rely on cross-sectional household survey data and cannot infer causality in observed associations. The sample frame for these surveys is limited to areas served by AWCs, thus, possibly, excluding some limited areas in the state. Women’s self-reported information in surveys is subject to social desirability and recall bias—the former would bias our associations upwards by causing women with more exposure to FHW advice to over-report positive behaviours; the latter would only affect our findings if differential, making associations of interest appear stronger. As the case with most studies, there is risk for residual confounding. Programme components that may have played a role in the observed associations, such as mass-media education campaigns, interactions with FHWs and other health providers during immunisation campaigns and VHSND, were not measured in household surveys in Bihar. Of note, our ‘falsification test’ worked well and gives confidence in our results—the only significant association between our control outcome (ie, TV ownership) and FHW exposure relates to the quality of interactions with FHWs, which can be explained by women being receptive to various channels of information. Importantly, given the tautological nature of the relationship between JSY programme participation and institutional deliveries in India, it is difficult to assess the true contribution of the FHW programme to changes in institutional deliveries during the period of study. A sensitivity analysis that adjusted our facility delivery models for JSY participation showed a significant effect of this programme on women delivering in a facility (online supplemental table 4).

## Conclusion

Implementation of FHW interventions should be continuously and strategically evaluated and adapted in Bihar. Attention should be given to addressing potential barriers to obtaining sustainable impact at scale, including weak leadership and supervision, limited implementation management capacity and variable engagement, competition between programme components, variation in implementation fidelity and FHW motivation. The relative costs and benefits of supporting FHW programmes alone or in tandem with other strategies to improve MNH outcomes should be considered. Adoption of a stepwise approach to FHW programme scale-up and integration into the health system and a thorough preparedness prior to transitioning to a technical support model for implementing and maintaining programmes at scale appear to be key to achieving positive and sustainable results.^10 44^

## Data Availability

Data are available upon request. Data can be made available upon request and under a data use agreement with CARE, India.
