# Supplementary material for: From direct engagement to technical support: a programmatic evolution to improve large community health worker programs in Bihar, India
Source: BMJ Glob Health. 2021 Apr 14;6(4):e004389. doi: 10.1136/bmjgh-2020-004389 (PMC8054080; doi:10.1136/bmjgh-2020-004389)
Supplement: Supplementary data [file bmjgh-2020-004389supp001.pdf]

e-Table 1. Program phases, data collection timing and data linkages

Program		Household Surveys			Facility Assessments
**Phase**	**Districts**	**Round**	**Date collection**	**Child age-groups**	**Date collection & linkages**
**IFHI**	**8 Districts** Begusarai, East Champaran, Gopalganj, Khagaria, Patna, Purnia, Rohtas, West Champaran	B[1]	Dec 2011 – Feb 2012	0-2 months	Not used in analysis
		P1	Oct 2012 – Dec 2012	0-2 months	April 2015– June 2015
		P2	Feb 2013 - Apr 2013	0-2 months	
		P3	June 2013 – Aug 2013	0-2 months	
		P4	Oct 2013 – Dec 2013	0-2 months	
**TSU**	**All 38 Districts**	S1	May 2014 – Sep 2014	0-2 months, 12-23 months	
		S2	Sept 2015 – Dec 2015	0-2 months, 12-23 months	
		S3	Oct 2016 – Dec 2016	0-2 months, 12-23 months	July 2016– Aug 2016[2]
		S4	Oct 2017 – Dec 2017	0-2 months 12-23 months	

***Notes*:** IFHI, Integrated Family Health Initiative; P1-P4, data round during IFHI phase; S1-S4, data round during TSU phase; TSU, Technical Support Unit.

[1]First survey round was not used due to data quality issues;
[2]Data from the second round of facility assessments used for S1-S4 models in Table 3.

e-Table 2. Frontline health worker visitation coverage and intensity

		Initial 8 program districts								Additional 30 districts				All 38 districts			
**Program outputs**		**P1**	**P2**	**P3**	**P4**	**S1**	**S2**	**S3**	**S4**	**S1**	**S2**	**S3**	**S4**	**S1**	**S2**	**S3**	**S4**
		Weighted proportion (95% CI)															
**Frontline health worker program**	Any visit during pregnancy	0.59 (0.57-0.61)	0.57 (0.55-0.59)	0.58 (0.56-0.60)	0.66 (0.64-0.68)	0.54 (0.52-0.55)	0.48 (0.46-0.50)	0.44 (0.42-0.46)	0.40 (0.38-0.41)	0.40 (0.39-0.41)	0.38 (0.37-0.39)	0.41 (0.40-0.42)	0.36 (0.35-0.37)	0.44 (0.43-0.44)	0.41 (0.40-0.42)	0.42 (0.41-0.43)	0.37 (0.36-0.37)
	Visits last 3 months of pregnancy (any)	0.59 (0.57-0.61)	0.56 (0.54-0.58)	0.57 (0.55-0.59)	0.64 (0.62-0.66)	0.47 (0.45-0.49)	0.44 (0.42-0.46)	0.38 (0.36-0.40)	0.32* (0.30-0.34)	0.37 (0.36-0.38)	0.35 (0.34-0.36)	0.35 (0.34-0.36)	0.30^ (0.29-0.30)	0.40 (0.39-0.40)	0.38 (0.378-0.38)	0.36 (0.35-0.36)	0.30 (0.29-0.31)
	0	0.41 (0.39-0.43)	0.44 (0.42-0.46)	0.43 (0.41-0.45)	0.36 (0.34-0.38)	0.52 (0.50-0.54)	0.59 (0.57-0.61)	0.64 (0.62-0.66)	0.68* (0.66-0.70)	0.62 (0.61-0.63)	0.67 (0.66-0.68)	0.66 (0.65-0.67)	0.71^ (0.70-0.72)	0.59 (0.58-0.60)	0.65 (0.64-0.66)	0.66 (0.65-0.67)	0.70 (0.69-0.71)
	1	0.07 (0.06-0.08)	0.05 (0.04-0.06)	0.06 (0.05-0.07)	0.04 (0.03-0.05)	0.07 (0.06-0.07)	0.11 (0.10-0.128)	0.08 (0.07-0.09)	0.08 (0.07-0.09)	0.05 (0.05-0.06)	0.09 (0.09-0.10)	0.07 (0.07-0.08)	0.07^ (0.06-0.07)	0.06 (0.05-0.06)	0.10 (0.09-0.10)	0.08 (0.07-0.08)	0.07 (0.07-0.07)
	2	0.11 (0.10-0.13)	0.11 (0.10-0.13)	0.19 (0.18-0.21)	0.14 (0.13-0.168)	0.12 (0.11-0.14)	0.11 (0.10-0.12)	0.08 (0.07-0.09)	0.07* (0.06-0.08)	0.09 (0.09-0.10)	0.09 (0.08-0.09)	0.07 (0.079-0.089)	0.07^ (0.07-0.08)	0.10 (0.10-0.11)	0.09 (0.09-0.10)	0.08 (0.07-0.08)	0.07 (0.07-0.07)
	3+	0.40 (0.38-0.42)	0.40 (0.38-0.42)	0.32 (0.30-0.34)	0.46 (0.44-0.48)	0.29 (0.28-0.31)	0.19 (0.18-0.21)	0.20 (0.18-0.21)	0.17* (0.16-0.19)	0.24 (0.23-0.25)	0.15 (0.15-0.16)	0.19 (0.18-0.20)	0.16^ (0.15-0.16)	0.25 (0.25-0.26)	0.16 (0.16-0.17)	0.19 (0.16-020)	0.16 (0.16-0.17)
	Visits first week postpartum (any)	0.43 (0.41-0.45)	0.48 (0.46-0.50)	0.49 (0.47-0.51)	0.56 (0.54-0.58)	0.36 (0.36-0.38)	0.38 (0.37-0.40)	0.39 (0.37-0.41)	0.45 (0.43-0.57)	0.31 (0.31-0.33)	0.36 (0.36-0.37)	0.35 (0.34-0.36)	0.40^ (0.39-0.41)	0.33 (0.32-0.34)	0.37 (0.36-0.38)	0.36 (0.35-0.37)	0.32 (0.31-0.33)
	0	0.57 (0.55-0.59)	0.52 (0.50-0.54)	0.51 (0.49-0.53)	0.44 (0.42-0.46)	0.64 (0.62-0.65)	0.62 (0.60-0.63)	0.61 (0.59-0.63)	0.55 (0.53-0.56)	0.69 (0.68-0.70)	0.64 (0.63-0.65)	0.65 (0.64-0.66)	0.60^ (0.59-0.61)	0.67 (0.67-0.68)	0.63 (0.62-0.64)	0.64 (0.63-0.65)	0.58 (0.57-0.59)
	1	0.05 (0.04-0.06)	0.10 (0.09-0.11)	0.10 (0.09-0.11)	0.11 (0.10-0.12)	0.11 (0.10-0.12)	0.18 (0.17-0.19)	0.17 (0.15-0.18)	0.22* (0.21-0.23)	0.11 (0.10-0.11)	0.18 (0.17-0.19)	0.14 (0.14-0.15)	0.19^ (0.19-0.20)	0.11 (0.10-0.11)	0.18 (0.17-0.19)	0.15 (0.14-0.16)	0.20 (0.19-0.21)
	2+	0.39 (0.37-0.41)	0.38 (0.36-0.40)	0.39 (0.37-0.41)	0.45 (0.43-0.47)	0.25 (0.24-0.27)	0.21 (0.19-0.22)	0.22 (0.21-0.24)	0.24* (0.22-0.25)	0.21 (0.20-0.22)	0.18 (0.18-0.19)	0.21 (0.20-0.22)	0.21 (0.20-0.22)	0.22 (0.21-0.23)	0.19 (0.18-0.20)	0.21 (0.21-0.22)	0.22 (0.21-0.22)
	Advice on pregnancy danger signs	0.17 (0.157-0.187)	0.18 (0.16-0.19)	0.18 (0.17-0.20)	0.27 (0.25-0.29)	0.11 (0.10-0.12)	0.05 (0.05-0.06)	0.11 (0.10-0.12)	0.11 (0.10-0.13)	0.05 (0.05-0.06)	0.03 (0.03-0.04)	0.09 (0.09-0.10)	0.08^ (0.08-0.09)	0.07 (0.06-0.07)	0.04 (0.04-0.04)	0.10 (0.09-0.10)	0.09 (0.09-0.10)

Wilhelm J, *et al. BMJ Global Health* 2022; 6:e004389. doi: 10.1136/bmjgh-2020-004389

	Advice to deliver in a health facility	0.47 (0.45-0.49)	0.51 (0.49-0.53)	0.48 (0.46-0.50)	0.57 (0.55-0.59)	0.42 (0.40-0.43)	0.43 (0.42-0.45)	0.33 (0.32-0.35)	0.38* (0.36-0.40)	0.33 (0.32-0.34)	0.35 (0.34-0.36)	0.32 (0.31-0.33)	0.34 (0.33-0.35)	0.35 (0.34-0.36)	0.37 (0.37-0.38)	0.32 (0.31-0.33)	0.35 (0.35-0.36)
	Advice to deliver with skilled birth attendant	0.16 (0.15-0.18)	0.16 (0.15-0.18)	0.18 (0.17-0.20)	0.20 (0.18-0.22)	0.09 (0.078-0.10)	0.04 (0.03-0.05)	0.08 (0.07-0.09)	0.09* (0.08-0.10)	0.05 (0.05-0.05)	0.02 (0.02-0.02)	0.06 (0.05-0.06)	0.06^ (0.05-0.06)	0.06 (0.06-0.06)	0.03 (0.02-0.03)	0.06 (0.06-0.07)	0.07 (0.06-0.07)
	Advice regarding safe delivery practices[1]	0.40 (0.45-0.49)	0.49 (0.46-0.51)	0.50 (0.48-0.52)	0.59 (0.57-0.61)	0.39 (0.37-0.40)	0.31 (0.30-0.33)	0.28 (0.26-0.30)	0.30* (0.29-0.32)	0.26 (0.25-0.27)	0.20 (0.19-0.21)	0.23 (0.22-0.24)	0.24^ (0.23-0.25)	0.29 (0.29-0.30)	0.23 (0.22-0.24)	0.24 (0.24-0.25)	0.26 (0.25-0.27)
	Advice regarding family planning	0.21 (0.19-0.22)	0.22 (0.21-0.24)	0.23 (0.21-0.24)	0.33 (0.31-0.35)	0.17 (0.16-0.19)	0.12 (0.11-0.13)	0.14 (0.13-0.15)	0.15* (0.14-0.16)	0.12 (0.11-0.13)	0.09 (0.08-0.09)	0.12 (0.12-0.13)	0.12 (0.12-0.13)	0.13 (0.19-0.14)	0.10 (0.09-0.10)	0.13 (0.12-0.13)	0.13 (0.12-0.14)
	Advice regarding skin-to-skin practice	0.14 (0.13-0.16)	0.17 (0.16-0.19)	0.17 (0.15-0.18)	0.22 (0.20-0.23)	0.14 (0.13-0.16)	0.09 (0.08-0.10)	0.14 (0.13-0.16)	0.16 (0.15-0.17)	0.06 (0.06-0.06)	0.04 (0.03-0.04)	0.10 (0.10-0.11)	0.11^ (0.10-0.11)	0.08 (0.08-0.09)	0.05 (0.05-0.06)	0.11 (0.11-0.12)	0.12 (0.12-0.13)
	Advice regarding breastfeeding	0.42 (0.40-0.44)	0.44 (0.42-0.46)	0.46 (0.44-0.48)	0.54 (0.52-0.56)	0.33 (0.31-0.34)	0.26 (0.24-0.27)	0.24 (0.23-0.26)	0.22* (0.21-0.24)	0.22 (0.21-0.23)	0.17 (0.16-0.17)	0.19 (0.18-0.20)	0.18^ (0.17-0.18)	0.25 (0.24-0.26)	0.19 (0.18-0.20)	0.21 (0.20-0.21)	0.19 (0.18-0.19)

Notes: IFHI, Integrated Family Health Initiative; P1-P4, data round during IFHI phase; S1-S4, data round during TSU phase; TSU, Technical Support Unit. *Denotes statistically significant difference when comparing S4 vs. P1 at p<0.05; ^Denotes statistically significant difference when comparing S4 vs. S1 at p<0.05.

[1]Handwashing, cord care, drying, skin-to-skin contact, early initiation/exclusive breastfeeding.

Wilhelm J, *et al. BMJ Global Health* 2022; 6:e004389. doi: 10.1136/bmjgh-2020-004389

e-Table 3. Program performance on key maternal and neonatal outcomes

Outcome indicators		Initial 8 program districts								Additional 30 districts				All 38 districts			
		P1	P2	P3	P4	S1	S2	S3	S4	S1	S2	S3	S4	S1	S2	S3	S4
		Weighted proportion (95% CI)															
Maternal*	Antenatal care (any visit)	0.83 (0.81-0.84)	0.87 (0.85-0.88)	0.94 (0.93-0.95)	0.96 (0.95-0.97)	0.81 (0.79-0.82)	0.97 (0.96-0.97)	0.96 (0.96-0.97)	0.98* (0.97-0.98)	0.84 (0.84-0.85)	0.98 (0.97-0.98)	0.97 (0.97-0.98)	0.98^ (0.97-0.98)	0.83 (0.83-0.84)	0.97 (0.97-0.98)	0.97 (0.97-0.97)	0.98 (0.97-0.98)
	3+ visits	0.23 (0.22-0.25)	0.21 (0.20-0.23)	0.21 (0.20-0.23)	0.23 (0.22-0.25)	0.20 (0.19-0.22)	0.47 (0.45-0.48)	0.47 (0.45-0.49)	0.50* (0.48-0.52)	0.19 (0.18-0.20)	0.48 (0.47-0.49)	0.50 (0.49-0.51)	0.56^ (0.55-0.57)	0.19 (0.19-0.20)	0.48 (0.47-0.48)	0.50 (0.49-0.50)	0.54 (0.53-0.54)
	4+ visits	0.10 (0.09-0.11)	0.09 (0.08-0.10)	0.11 (0.10-0.12)	0.10 (0.09-0.12)	0.10 (0.09-0.11)	0.26 (0.24-0.27)	0.27 (0.26-0.29)	0.28* (0.27-0.30)	0.08 (0.07-0.08)	0.26 (0.25-0.27)	0.28 (0.27-0.29)	0.31^ (0.30-0.32)	0.08 (0.08-0.09)	0.26 (0.25-0.26)	0.28 (0.27-0.29)	0.30 (0.39-0.30)
	Facility delivery (all facilities)	0.70 (0.68-0.72)	0.71 (0.07-0.73)	0.75 (0.73-0.77)	0.74 (0.72-0.75)	0.75 (0.73-0.76)	0.75 (0.74-0.77)	0.74 (0.72-0.75)	0.79* (0.78-0.80)	0.71 (0.70-0.72)	0.69 (0.68-0.70)	0.69 (0.68-0.70)	0.75 (0.74-0.76)	0.72 (0.71-0.73)	0.71 (0.70-0.71)	0.70 (0.70-0.71)	0.76 (0.75-0.77)
	Public facility delivery	0.58 (0.56-0.60)	0.59 (0.57-0.61)	0.62 (0.60-0.64)	0.62 (0.60-0.64)	0.60 (0.59-0.62)	0.59 (0.57-0.61)	0.57 (0.55-0.59)	0.63* (0.61-0.64)	0.56 (0.55-0.57)	0.53 (0.52-0.54)	0.53 (0.52-0.54)	0.58^ (0.57-0.59)	0.57 (0.56-0.58)	0.54 (0.54-0.55)	0.54 (0.53-0.55)	0.59 (0.58-0.60)
	Private facility delivery	0.12 (0.11-0.13)	0.13 (0.11-0.14)	0.13 (0.12-0.14)	0.12 (0.11-0.13)	0.14 (0.13-0.16)	0.16 (0.15-0.18)	0.16 (0.15-0.18)	0.16* (0.15-0.18)	0.15 (0.14-0.15)	0.16 (0.16-0.17)	0.16 (0.15-0.17)	0.17 (0.16-0.18)	0.15 (0.14-0.15)	0.16 (0.16-0.17)	0.16 (0.16-0.17)	0.17 (0.16-0.17)
	Skilled birth attendant if home delivery	0.04 (0.03-0.06)	0.05 (0.03-0.06)	0.06 (0.04-0.08)	0.04 (0.03-0.06)	0.04 (0.03-0.06)	0.03 (0.02-0.04)	0.02 (0,01-0.03)	0.02* (0.01-0.03)	0.06 (0.05-0.07)	0.03 (0.03-0.04)	0.03 (0.03-0.04)	0.03^ (0.03-0.04)	0.05 (0.05-0.06)	0.03 (0.03-0.04)	0.03 (0.03-0.04)	0.03 (0.02-0.04)
Neonatal**	Skin-to-skin right after birth (all deliveries)	0.14 (0.13-0.16)	0.16 (0.15-0.18)	0.17 (0.15-0.18)	0.24 (0.22-0.25)	0.17 (0.16-0.18)	0.11 (0.1-0.13)	0.19 (0.18-0.20)	0.19* (0.18-0.21)	0.11 (0.10-0.12)	0.06 (0.06-0.07)	0.17 (0.17-0.18)	0.17^ (0.16-0.17)	0.13 (0.12-0.13)	0.08 (0.07-0.08)	0.18 (0.17-0.18)	0.17 (0.17-0.18)
	Public facility delivery	0.18 (0.16-0.20)	0.20 (0.18-0.22)	0.20 (0.18-0.22)	0.29 (0.27-0.32)	0.22 (0.21-0.24)	0.16 (0.14-0.17)	0.26 (0.24-0.29)	0.21* (0.20-0.23)	0.15 (0.14-0.16)	0.10 (0.09-0.10)	0.25 (0.24-0.26)	0.18^ (0.17-0.19)	0.17 (0.16-0.18)	0.11 (0.11-0.12)	0.26 (0.25-0.27)	0.23 (0.22-0.24)
	Private facility delivery	0.10 (0.07-0.15)	0.13 (0.09-0.17)	0.13 (0.1-0.18)	0.17 (0.13-0.22)	0.13 (0.1-0.16)	0.06 (0.04-0.08)	0.11 (0.08-0.14)	0.09 (0.06-0.11)	0.08 (0.07-0.09)	0.03 (0.03-0.04)	0.09 (0.08-0.11)	0.10^ (0.08-0.11)	0.09 (0.08-0.11)	0.04 (0.03-0.05)	0.10 (0.08-0.11)	0.09 (0.08-0.11)
	Home delivery	0.07 (0.06-0.10)	0.10 (0.08-0.13)	0.09 (0.07-0.12)	0.14 (0.11-0.17)	0.06 (0.05-0.08)	0.05 (0.04-0.07)	0.08 (0.06-0.10)	0.08 (0.06-0.10)	0.05 (0.04-0.05)	0.02 (0.02-0.03)	0.08 (0.07-0.09)	0.08^ (0.07-0.10)	0.05 (0.04-0.06)	0.03 (0.03-0.04)	0.08 (0.07-0.09)	0.08 (0.07-0.09)

	Early initiation of breastfeeding (all deliveries)	0.50 (0.48-0.52)	0.53 (0.51-0.55)	0.63 (0.61-0.65)	0.59 (0.57-0.61)	0.61 (0.60-0.63)	0.77 (0.75-0.78)	0.63 (0.61-0.65)	0.72* (0.70-0.74)	0.55 (0.54-0.56)	0.75 (0.74-0.75)	0.58 (0.57-0.59)	0.69^ (0.68-0.70)	0.57 (0.56-0.58)	0.75 (0.74-0.76)	0.59 (0.59-0.6)	0.70 (0.69-0.71)
	Public facility delivery	0.80 (0.77-0.83)	0.73 (0.70-0.75)	0.74 (0.72-0.77)	0.81 (0.78-0.83)	0.83 (0.81-0.85)	0.84 (0.82-0.86)	0.81 (0.79-0.83)	0.82 (0.81-0.84)	0.77 (0.76-0.78)	0.80 (0.79-0.81)	0.78 (0.77-0.79)	0.81^ (0.80-0.82)	0.79 (0.78-0.80)	0.81 (0.8-0.82)	0.79 (0.78-0.8)	0.82 (0.81-0.83)
	Private facility delivery	0.77 (0.70-0.82)	0.56 (0.49-0.62)	0.61 (0.54-0.67)	0.43 (0.36-0.50)	0.52 (0.47-0.58)	0.67 (0.63-0.71)	0.48 (0.42-0.53)	0.48* (0.43-0.54)	0.52 (0.49-0.55)	0.70 (0.68-0.72)	0.42 (0.39-0.45)	0.48^ (0.45-0.51)	0.52 (0.49-0.55)	0.69 (0.67-0.71)	0.44 (0.41-0.46)	0.48 (0.45-0.51)
	Home delivery	0.77 (0.73-0.81)	0.54 (0.50-0.58)	0.61 (0.57-0.65)	0.62 (0.57-0.66)	0.56 (0.52-0.60)	0.67 (0.64-0.70)	0.45 (0.42-0.49)	0.53* (0.49-0.57)	0.59 (0.57-0.61)	0.68 (0.66-0.70)	0.45 (0.43-0.46)	0.49^ (0.47-0.51)	0.58 (0.56-0.6)	0.68 (0.66-0.69)	0.45 (0.43-0.46)	0.50 (0.48-0.52)
	Newborn weighed at birth (all deliveries)	0.52 (0.50-0.54)	0.57 (0.55-0.59)	0.63 (0.61-0.65)	0.62 (0.60-0.64)	0.64 (0.62-0.66)	0.68 (0.66-0.70)	0.66 (0.64-0.68)	0.73* (0.71-0.74)	0.62 (0.62-0.63)	0.63 (0.63-0.64)	0.66 (0.65-0.66)	0.71^ (0.70-0.72)	0.63 (0.62-0.64)	0.65 (0.64-0.66)	0.66 (0.65-0.66)	0.71 (0.71-0.72)
	Public facility delivery	0.74 (0.72-0.76)	0.77 (0.75-0.79)	0.82 (0.8-0.84)	0.84 (0.82-0.86)	0.91 (0.90-0.92)	0.95 (0.94-0.96)	0.95 (0.93-0.96)	0.92* (0.91-0.93)	0.90 (0.90-0.91)	0.93 (0.93-0.94)	0.95 (0.95-0.96)	0.93^ (0.92-0.94)	0.91 (0.90-0.91)	0.94 (0.93-0.94)	0.95 (0.95-0.96)	0.93 (0.92-0.93)
	Private facility delivery	0.59 (0.53-0.64)	0.68 (0.62-0.73)	0.72 (0.66-0.77)	0.63 (0.57-0.69)	0.73 (0.68-0.77)	0.71 (0.67-0.75)	0.74 (0.69-0.78)	0.72* (0.68-0.76)	0.77 (0.75-0.79)	0.76 (0.74-0.78)	0.78 (0.76-0.8)	0.76 (0.74-0.78)	0.76 (0.74-0.78)	0.75 (0.73-0.77)	0.77 (0.75-0.79)	0.75 (0.73-0.77)
	Home delivery	0.07 (0.05-0.09)	0.12 (0.09-0.14)	0.09 (0.07-0.11)	0.10 (0.08-0.13)	0.07 (0.06-0.09)	0.10 (0.08-0.12)	0.10 (0.08-0.12)	0.13 (0.11-0.16)	0.11 (0.09-0.12)	0.11 (0.10-0.13)	0.14 (0.13-0.15)	0.15^ (0.14-0.17)	0.10 (0.09-0.11)	0.11 (0.1-0.12)	0.13 (0.12-0.14)	0.15 (0.13-0.16)

Notes: IFHI, Integrated Family Health Initiative; P1-P4, data round during IFHI phase; S1-S4, data round during TSU phase; TSU, Technical Support Unit. *Denotes statistically significant difference when comparing S4 vs. P1 at p<0.05; ^Denotes statistically significant difference when comparing S4 vs. S1 at p<0.05.

e-Table 4. Sensitivity analysis -- Results from multivariate regression models fitted for institutional delivery models also adjusted for Janani Suraksha Yojana participation

Covariates	IFHI and TSU phase data in 8 initial districts	TSU phase data in all 38 districts
	Adjusted-OR (95% Confidence Interval)	
Socio-demographic characteristics		
Age	1.014* (1.003,1.025)	1.008* (1.001,1.014)
Parity	0.825*** (0.802,0.849)	0.837*** (0.823,0.851)
Non-Hindu religion (Hindu=ref)	0.830*** (0.748,0.921)	0.731*** (0.690,0.775)
Marginalized caste (Other=ref)	0.658*** (0.608,0.712)	0.587*** (0.561,0.613)
Middle wealth index tertile (High index tertile=ref)	1.076 (0.991,1.169)	1.063* (1.014,1.114)
Low index tertile	1.179*** (1.083,1.284)	1.077** (1.028,1.129)
Woman’s literacy (No=ref)	1.774*** (1.634,1.926)	1.629*** (1.556,1.706)
Husband’s literacy (No=ref)	1.314*** (1.220,1.416)	1.324*** (1.268,1.382)
Context Variables		
JSY program participation (no=ref)	77.99*** (53.24,114.25)	20.17*** (17.14,23.74)
TSU phase (IFHI=ref)	1.910*** (1.730,2.110)	
Frontline health worker program outputs		
1+ FHW household visits (No visit=ref)	1.389*** (1.245,1.549)	
Interaction term for 1+ visits & program phase	1.185* (1.029,1.364)	
Facility factors		
First referral unit facility level (Primary health center=ref)	1.172 (0.944,1.455)	1.082 (0.948,1.236)
District hospital	1.050 (0.872,1.265)	1.247** (1.091,1.425)
Clinical staff filled: approved ratio (Fully staffed=ref)	1.099** (1.032,1.171)	1.132*** (1.087,1.179)
Constant	1.033 (0.789,1.352)	1.702*** (1.456,1.989)

Notes: FHW, frontline health worker; IFHI, Integrated Family Health Initiative; JSY, Janani Suraksha Yojana; TSU, Technical Support Unit. Models adjusted for all the factors shown. *Denotes adjusted-ORs statistically significant at *p<0.05; **p<0.01; or ***p<0.001.

e-Figure 1. Distribution of the quality index for interactions with frontline health workers

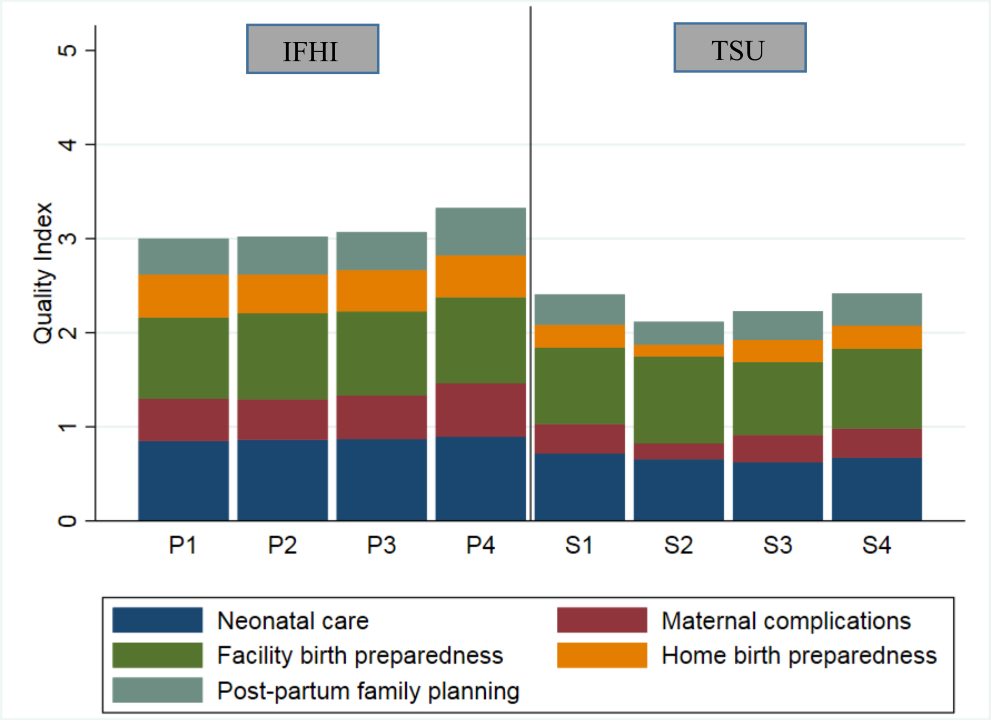

Notes: IFHI, Integrated Family Health Initiative; P1-P4, data round during IFHI phase; S1-S4, data round during TSU phase; TSU, Technical Support Unit. The quality index was estimated among mothers of 0-2 month old infants who received one or more frontline health worker visits during pregnancy. The index accounts for the number of domains of advice received across five domains: birth preparedness for institutional delivery, birth preparedness for home delivery (whether planned or unplanned), recognition and care seeking for maternal complications, essential newborn care practices, and postpartum family planning. The index ranges between 0 and 5. For each survey, types of advice are shown as proportions of women receiving advice in each domain and displayed as stacked bars to illustrate the relative contribution of each domain to the mean index score. Proportions are weighted.

e-Figure 2. Trends in Janani Suraksha Yojana program participation and institutional delivery by survey round

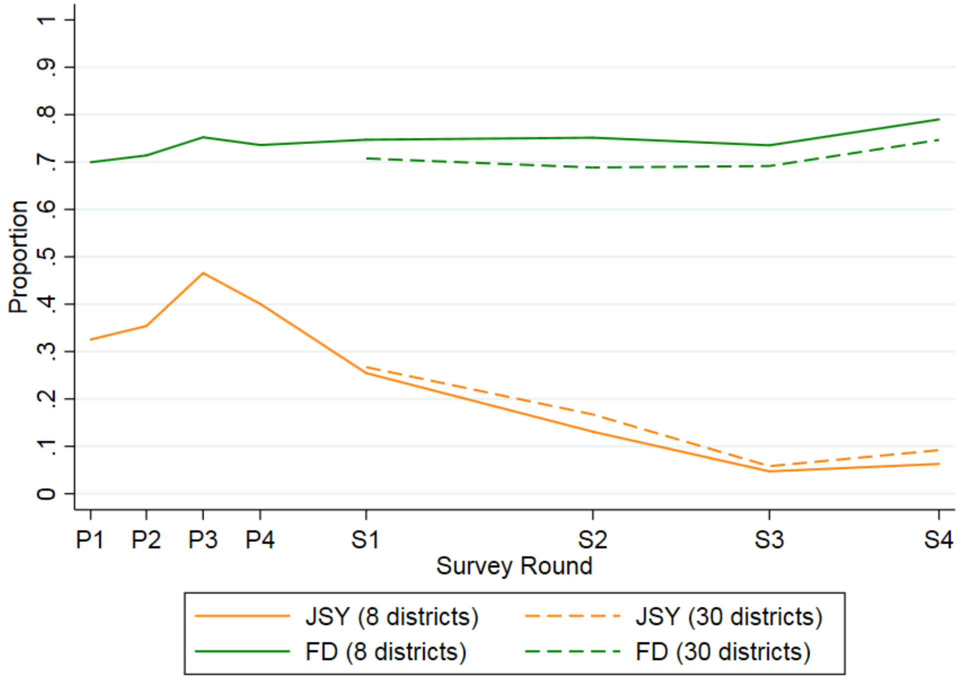

Notes: FD, institutional delivery; JSY, Janani Suraksha Yojana.
